# Prognostic value of objective nutritional indices (PNI, CONUT, and HALP) for in-hospital all-cause mortality in surgically treated infective endocarditis: a 14-year retrospective study in a Chinese cohort

**DOI:** 10.3389/fnut.2026.1840852

**Published:** 2026-07-01

**Authors:** Guangxu Mao, Ziyao Quan, Liyun Wang, Feng Zang

**Affiliations:** 1 Department of Infection Management, Xinghua People's Hospital Affiliated to Yangzhou University, Xinghua, Jiangsu, China; 2 Department of Infection Management, General Hospital of Eastern Theater Command, People’s Liberation Army, Nanjing, Jiangsu, China; 3 Department of Infection Management, The First Affiliated Hospital with Nanjing Medical University, Nanjing, Jiangsu, China; 4 Ili and Jiangsu Joint Institute of Health, Yining, Xinjiang, China; 5 The Friendship Hospital of Ili Kazakh Autonomous Prefecture, Yining, Xinjiang, China

**Keywords:** CONUT score, HALP score, infective endocarditis, in-hospital all-cause mortality, nutritional status, prognostic nutritional index

## Abstract

**Objective:**

Malnutrition is closely associated with poor clinical outcomes; however, its prognostic value in patients with infective endocarditis (IE) undergoing surgical treatment remains unclear. This study aimed to investigate the association of three objective nutritional indices (PNI, CONUT, and HALP) with in-hospital all-cause mortality in surgically treated patients with IE.

**Methods:**

In this retrospective study, clinical data were collected from 450 patients with IE who underwent surgical treatment at Jiangsu Provincial People’s Hospital over 14 years. Patients were categorized into a non-survivor group (*n* = 42) and a survivor group (*n* = 408). Nutritional status was assessed using the PNI, CONUT, and HALP scores. Stepwise-adjusted multivariate logistic regression models and receiver operating characteristic (ROC) curve analyses were used to evaluate the independent prognostic value and discriminatory performance of these nutritional indices for in-hospital all-cause mortality.

**Results:**

The in-hospital all-cause mortality rate in this cohort was 9.33%. Preoperative malnutrition risk was common among patients with IE, with nutritional risk identified in 44.00 and 75.11% of patients according to the PNI and CONUT criteria, respectively. Compared with survivors, non-survivors had significantly poorer baseline nutritional status. After adjustment for confounding factors, each one-unit increase in the PNI was associated with a 7% lower risk of in-hospital all-cause mortality (OR = 0.93, 95% CI: 0.89–0.98, *p* = 0.009). Similarly, each one-unit increase in the HALP score was associated with a 4% lower risk of in-hospital all-cause mortality (OR = 0.96, 95% CI: 0.93–0.98, *p* = 0.001). In contrast, each one-unit increase in the CONUT score was associated with a 16% higher risk of in-hospital all-cause mortality (OR = 1.16, 95% CI: 1.03–1.32, *p* = 0.017). A prognostic model incorporating these nutritional indicators showed good discrimination (AUC = 0.839) and acceptable predictive accuracy (Brier score = 0.063).

**Conclusion:**

Preoperative malnutrition risk is highly prevalent among patients with IE undergoing surgery and is an independent predictor of postoperative in-hospital all-cause mortality. Routine use of objective nutritional scoring tools, including the PNI, CONUT, and HALP, may improve preoperative risk stratification. Early nutritional assessment and targeted intervention for patients at moderate-to-severe nutritional risk may provide meaningful clinical benefits.

## 1 Introduction

Infective endocarditis (IE) is a severe infection of the endocardium and valvular structures caused by pathogenic microbial colonization, often leading to marked systemic pathophysiological disturbances (1). Despite advances in antimicrobial therapy and surgical management, clinical outcomes in patients with IE remain suboptimal (2, 3). Epidemiological studies have reported inpatient mortality rates ranging from 6 to 30% (4, 5), mortality peaking at 25 to 30% at 6 months (6), and an approximately 40% mortality rate at 1 year (7, 8). Large global burden-of-disease analyses have also shown increasing age-standardized incidence and mortality over the past three decades (9, 10). These trends suggest that current standards of care do not fully offset the overall disease burden, making it important to identify additional prognostic determinants and potentially modifiable risk factors.

Malnutrition and cachexia are common but often underrecognized comorbidities in severe systemic infection. The profound systemic inflammatory response triggered by IE can accelerate catabolism while weakening immune competence, creating a pathological cycle of nutritional depletion, immune dysfunction, and progressive infectious deterioration (11). Timely nutritional assessment and targeted intervention may help improve clinical management in high-risk patients. Objective nutritional indices widely used in prognostic risk stratification across cardiovascular and intensive care settings include the Prognostic Nutritional Index (PNI), the Controlling Nutritional Status (CONUT) score, and the Hemoglobin-Albumin-Lymphocyte-Platelet (HALP) score. Compared with single biomarkers, these composite indices may better reflect the interaction between immunonutritional depletion and systemic inflammation (12, 13).

The rationale for evaluating these composite nutritional indices in IE is supported by their established prognostic value in broader cardiovascular populations. Recent comparative studies have shown that these scoring systems have meaningful predictive ability in cardiovascular cohorts. For example, a lower HALP score has been reported as an independent predictor of 1-year and 5-year all-cause mortality in patients with non-valvular atrial fibrillation (NVAF) (14). Comparative analyses of CONUT, PNI, and the Geriatric Nutritional Risk Index in NVAF populations have further supported the association between malnutrition and adverse clinical outcomes, with CONUT showing value for long-term risk stratification (15). In addition, nutritional and systemic inflammatory status, assessed using HALP, CONUT, and PNI, has been linked to cardiac electrophysiological balance, as reflected by the corrected Index of Cardiac Electrophysiological Balance (ICEBc) (16). Together, these findings provide a clinical and pathophysiological basis for testing the prognostic utility of nutritional indices in the severe inflammatory setting of IE.

Although these composite indices have shown value in broader cardiovascular contexts, evidence in IE cohorts remains limited, and their prognostic role has not been fully defined. Therefore, this study aimed to evaluate and compare the predictive value of PNI, CONUT, and HALP scores for in-hospital all-cause mortality in surgically treated patients with IE. By comparing these indices, we sought to provide an evidence-based basis for early risk stratification and more individualized, patient-centered nutritional management.

## 2 Materials and methods

### 2.1 Study design and sample

**Figure 1:**
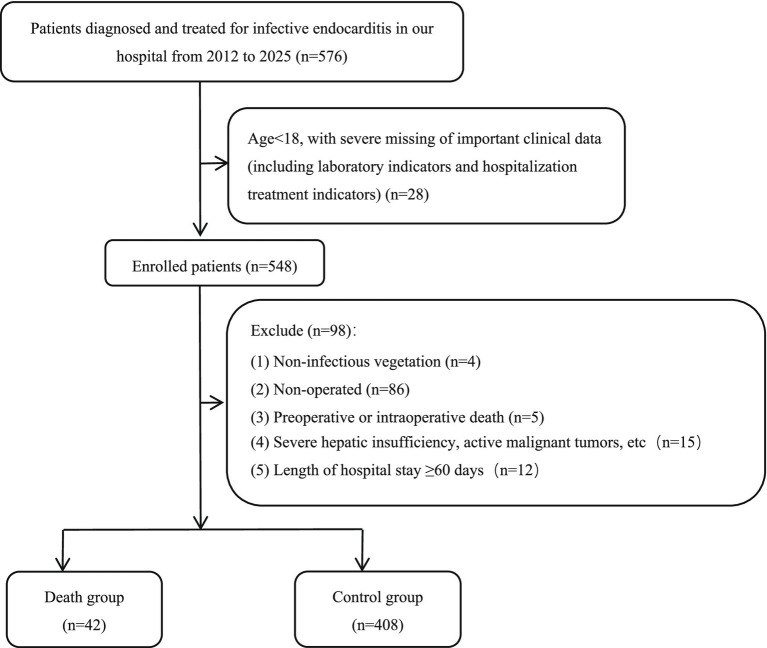
Participant flow diagram.

A retrospective analysis was conducted using clinical data from 450 patients diagnosed with IE who underwent surgical treatment at Jiangsu Provincial People’s Hospital between 2012 and 2025. Using in-hospital all-cause mortality as the endpoint, the cohort was divided into a non-survivor group and a survivor group (Figure 1). The inclusion criteria were as follows: (1) diagnosis of IE based on the modified Duke criteria from the 2015 ESC Guidelines on Endocarditis (5); (2) surgical treatment for IE; and (3) age 18 years or older. The exclusion criteria were: (1) non-infectious vegetations; (2) death before or during surgery; (3) severe systemic comorbidities likely to affect nutritional assessment and clinical outcomes, such as hematological malignancies, systemic autoimmune diseases (e.g., systemic lupus erythematosus or rheumatoid arthritis), advanced solid tumors, or long-term systemic immunosuppressive therapy (e.g., corticosteroids or chemotherapy); (4) incomplete key clinical data, including laboratory parameters and in-hospital treatment indicators; and (5) excessively prolonged hospital stay (60 days or longer). Patients with a hospital stay of 60 days or longer were excluded to reduce the influence of extreme clinical outliers. From a statistical perspective, extreme length-of-stay values tend to be heavy-tailed outliers and may affect the performance and generalizability of prognostic models (17, 18). Clinically, this study focused on the predictive value of baseline nutritional indices measured within 24 h of admission. In patients requiring hospitalization for more than 2 months, outcomes are often driven by downstream refractory nosocomial events, such as severe hospital-acquired infection or prolonged multiorgan failure. In these cases, baseline nutritional scores may no longer adequately reflect the patient’s condition at the clinical endpoint. Therefore, excluding these outliers helped maintain a more homogeneous study population.

PASS 15.0 software was used to estimate the required sample size based on the expected in-hospital all-cause mortality rate among patients with IE. Previous epidemiological studies indicate an expected in-hospital all-cause mortality rate of approximately 10%. With a 95% confidence level and a 3% margin of error, the minimum required sample size was 384 cases. Ultimately, 450 patients were included, with 42 endpoint events, providing sufficient statistical power for the planned analyses.

### 2.2 Data collection

#### 2.2.1 Perioperative data from patients diagnosed with IE were extracted from the institutional Health Information System (HIS), Laboratory Information System (LIS), and Picture Archiving and Communication System (PACS)

The collected variables included demographic characteristics, in-hospital treatment indicators, and laboratory results. Demographic data included age, sex, body mass index (BMI), smoking status, alcohol consumption, cardiac comorbidities (e.g., congenital heart disease and heart failure), and non-cardiac comorbidities (e.g., hypertension, diabetes mellitus, and chronic kidney disease). All comorbidities were retrospectively identified from electronic health records. Attending physicians documented confirmed diagnoses using International Classification of Diseases, 10th Revision (ICD-10) codes on the front page of each medical record, in accordance with Chinese national medical record standards. As this was a retrospective study, diagnoses were made according to the international guidelines applicable at the time of each admission. Heart failure was diagnosed according to the prevailing ESC Guidelines on Heart Failure (19–21). Hypertension was defined according to the 2018 ESC/ESH Guidelines (≥140/90 mmHg) (22). Diabetes mellitus was diagnosed using ADA criteria (23). Chronic kidney disease was defined according to the 2012 KDIGO guidelines (24). Congenital heart disease was diagnosed according to the 2008 ACC/AHA (25) and 2020 ESC guidelines (26). BMI was analyzed according to the criteria defined by the Working Group on Obesity in China (WGOC) (27). In-hospital treatment indicators included duration of surgery, intensive care unit (ICU) length of stay, duration of postoperative antimicrobial treatment, hemodialysis, healthcare-associated infection, mechanical ventilation duration, and duration of catheterization, including central venous and urinary catheters. Standard laboratory parameters included hematological indices (e.g., leukocyte, lymphocyte, and neutrophil counts), biochemical indices (e.g., albumin, alanine aminotransferase, and aspartate aminotransferase), and coagulation parameters (e.g., prothrombin time, fibrinogen, and D-dimer). All laboratory analyses were performed using fasting blood samples obtained within 24 h of hospital admission.

Doppler echocardiography was performed within 48 h of admission. The following variables were extracted: LVEF (%), LVEDD (mm), maximal vegetation diameter, valvular stenosis (moderate or severe vs. none/mild), valvular regurgitation (moderate or severe vs. none/mild), valvular prolapse (yes/no), valvular perforation (yes/no), and perivalvular abscess (yes/no). According to the latest research, the maximum vegetation diameter is classified as <10 mm or ≥10 mm (28, 29).

#### 2.2.2 Preoperative nutritional evaluation

PNI, CONUT, and HALP scores (12, 30). The calculation formulas and evaluation criteria are shown in Table 1.

**Table 1:** Objective nutritional indices used in this study.

Nutritional indices	Calculation method
Prognostic nutritional index (PNI) score	albumin (g/L) + 5 × lymphocyte count (×10^9^/L)
PNI group	Normal: >38, Moderate: 35–38, Severe: <35
Haemoglobin-albumin-lymphocyte-platelet (HALP) score	[haemoglobin (g/L) × albumin (g/L) × lymphocytes (×10^9^/L)]/[platelet count (×10^9^/L)]
Controlling Nutritional Status (CONUT) score	albumin score + total cholesterol score + lymphocyte count score

	Normal	Mild	Moderate	Severe
Albumin (g/L)	≥35	30–34.9	25–29.99	<25
score	0	2	4	6
Lymphocytes count (×10^9^/L)	≥1.6	1.2–1.59	0.8–1.19	<0.8
score	0	1	2	3
Total cholesterol (mmol/L)	≥4.66	3.63–4.65	2.59–3.62	<2.59
score	0	1	2	3
Total score	0–1	2–4	5–12	

### 2.3 Statistical analyses

Data analysis was performed using SPSS version 31.0 and R software. Continuous variables are presented as mean ± standard deviation or median (interquartile range), as appropriate. If data were normally distributed, between-group comparisons were performed using the t-test; otherwise, the Mann–Whitney *U* test was used. Categorical variables are presented as frequency (percentage), and group comparisons were performed using the chi-square test or Fisher’s exact test. A *p*-value <0.05 was considered statistically significant.

To assess multicollinearity among variables, Spearman’s correlation analysis was performed for statistically significant covariates (*p* < 0.05) in the univariate analysis. A correlation coefficient *r* > 0.5 was considered to indicate a moderate linear relationship. The variance inflation factor (VIF) was also calculated, and variables with severe multicollinearity (VIF ≥ 10) were excluded from the multivariate analysis (31).

To evaluate the independent associations between the three nutritional indices and postoperative in-hospital all-cause mortality, three stepwise multivariate logistic regression models were constructed: Model 1 (unadjusted), Model 2 (adjusted for general demographic variables), and Model 3 (further adjusted for in-hospital treatment indicators). Forest plots were used to visualize the multivariate results. Receiver operating characteristic (ROC) curve analysis was performed to compare the discriminatory ability of individual nutritional indices and integrated prognostic models for in-hospital all-cause mortality. Predictive accuracy was assessed using the area under the ROC curve (AUC) and its 95% confidence interval (CI).

## 3 Results

### 3.1 Univariate analysis of in-hospital all-cause mortality in patients with IE

Among the 450 patients with IE who underwent surgical treatment, 42 died postoperatively during hospitalization, resulting in an in-hospital all-cause mortality rate of 9.33% (Table 2). Univariate analysis identified several baseline differences associated with in-hospital all-cause mortality. The median age of non-survivors was higher than that of survivors (57 vs. 51 years, *p* = 0.021). Current smoking and hypertension were also associated with in-hospital all-cause mortality. Regarding clinical and surgical characteristics, healthcare-associated infection, duration of catheterization (including mechanical ventilation, central venous catheterization, and urinary catheterization), ICU length of stay, and duration of surgery were significantly higher in non-survivors than in survivors. Significant differences were also observed in laboratory parameters, including hemoglobin, albumin, serum creatinine, and D-dimer levels (Table 3). No statistically significant differences were observed between the death group and the control group in any of the echocardiographic parameters (all *p* > 0.05) (Table 4).

**Table 2:** Demographic and clinical surgical characteristics of patients with IE.

Characteristics	Overall (*n* = 450)	Non-survivors (*n* = 42)	Survivors (*n* = 408)	*Z*/χ^2^/*t*	*p*-value
Age, years	51 (39, 60)	57 (46, 65)	51 (39, 59)	−2.306	0.021
Sex, male	312 (69.33)	33 (78.57)	279 (68.38)	1.859	0.173
BMI	24.45 ± 3.77	24.12 ± 3.27	25.48 ± 3.81	−0.597	0.551
Current smoker	109 (24.22)	16 (38.09)	93 (22.79)	4.857	0.028
Alcohol consumption	94 (20.89)	13 (30.95)	81 (19.85)	2.839	0.092
Time from symptom onset to presentation	30.0 (14.0, 60.0)	30.0 (10.0, 90.0)	30.0 (15.0, 60.0)	−0.085	0.932
Cardiac comorbidities	78 (17.33)	9 (21.42)	69 (16.91)	0.542	0.462
Non-cardiac comorbidities					
Pneumonia	66 (14.67)	10 (23.81)	56 (13.72)	3.049	0.079
Hypertension	99 (22.00)	16 (38.09)	83 (20.34)	6.993	0.008
Diabetes mellitus	39 (8.66)	4 (9.52)	35 (8.58)	0.043	0.836
Chronic kidney disease	17 (3.78)	3 (7.14)	14 (3.43)	1.443	0.230
All-cause treatments					
Healthcare-associated infection	81 (18.00)	20 (47.62)	61 (14.95)	27.533	<0.01
Renal replacement therapy	16 (3.55)	2 (4.76)	14 (3.43)	0.197	0.657
Duration of mechanical ventilation	1.0 (1.0, 2.0)	3.0 (1.0, 10.0)	1.0 (1.0, 2.0)	−5.231	<0.001
Duration of the central venous catheter	5.0 (2.0, 9.0)	7.5 (3.7, 18.2)	5.0 (2.0, 9.0)	−2.781	<0.001
Duration of urinary catheterization (days)	5.0 (2.0, 9.0)	7.0 (3.7, 17.2)	5.0 (2.0, 9.0)	−2.624	<0.001
ICU length of stay (days)	2.0 (1.0, 6.0)	8.0 (3.7, 15.7)	2.0 (1.0, 5.0)	−5.590	<0.001
Duration of surgery (h)	4.8 (4.0, 5.8)	5.1 (4.1, 6.7)	4.7 (4.0, 5.7)	−1.521	<0.001
Elective surgery	207 (46.00)	24 (57.14)	183 (44.85)	2.315	0.128

**Table 3:** Laboratory parameters and nutritional assessment scores in patients with IE.

Laboratory parameters	Overall (*n* = 450)	Non-survivors (*n* = 42)	Survivors (*n* = 408)	*Z*/χ^2^	*p*-value
Hematologic parameters					
White blood cell (×10^9^/L)	8.1 (6.2, 11.0)	8.2 (6.0, 12.0)	8.0 (6.3, 10.7)	−0.490	0.624
Neutrophils (×10^9^/L)	6.1 (4.2, 8.8)	6.4 (5.1, 9.1)	6 (4.2, 8.7)	−1.298	0.194
Lymphocytes (×10^9^/L)	1.3 (0.9, 1.7)	1.1 (0.6, 1.7)	1.3 (0.9, 1.7)	−1.375	0.169
Hemoglobin (×10^9^/L)	105.0 (90.0, 118.2)	93.0 (82.5, 109.0)	106.0 (91.0, 119.0)	−2.579	0.010
Platelet (×10^9^/L)	178.0 (131.0, 241.2)	172.5 (129.0, 286.2)	178.0 (131.0, 240.0)	−0.466	0.641
Biochemical parameter					
Albumin (g/L)	32.9 (28.6, 36.8)	30.6 (26.6, 34.0)	33.3 (28.7, 37.0)	−2.666	0.008
ALT (U/L)	23.0 (13.1, 36.9)	22.4 (13.1, 29.5)	23.1 (13.0, 38.1)	−0.517	0.605
AST (U/L)	24.5 (18.4, 38.0)	22.2 (18.7, 41.0)	24.6 (18.2, 37.8)	−0.179	0.858
Total cholesterol (mmol/L)	3.4 (2.9, 4.1)	3.3 (2.4, 3.9)	3.4 (2.9, 4.2)	−1.726	0.084
Creatinine (μmol/L)	69.9 (56.9, 89.3)	88.1 (65.2, 121.0)	69.0 (56.3, 86.6)	−3.337	<0.001
TBIL (μmol/L)	11.0 (7.6, 15.4)	12.2 (8.6, 16.4)	10.9 (7.6, 15.4)	−0.983	0.326
DBIL (μmol/L)	4.6 (3.0, 6.5)	5.4 (3.2, 7.0)	4.5 (3.0, 6.4)	−1.477	0.140
Coagulation tests					
PT (s)	13.1 (12.3, 14.2)	13.1 (12.5, 14.4)	13.1 (12.3, 14.2)	−0.626	0.531
APTT (s)	30.5 (27.6, 34.2)	30.9 (28.8, 34.8)	30.5 (27.5, 34.1)	−1.444	0.149
TT (s)	17.1 (16.2, 18.1)	16.7 (15.9, 18.2)	17.2 (16.3, 18.0)	−1.390	0.165
Fibrinogen (g/L)	3.6 (2.8, 4.4)	3.4 (2.7, 4.2)	3.6 (2.9, 4.5)	−1.343	0.179
D-dimer (mg/L)	1.2 (0.6, 2.7)	2.6 (1.1, 3.9)	1.1 (0.6, 2.6)	−3.718	<0.001
Blood culture					
Negative	252 (56.00)	25 (59.52)	227 (55.63)	0.243	0.970
*Streptococcus* spp.	130 (28.89)	11 (26.19)	119 (29.16)		
*Staphylococcus* spp.	46 (10.22)	4 (9.52)	42 (10.29)		
Others	22 (4.89)	2 (4.76)	20 (4.90)		
Nutritional assessment scores					
PNI score	39.3 (34.5, 44.5)	35.9 (32.2, 40.9)	39.5 (34.6, 44.9)	−3.176	0.001
PNI classification					
Normal (>38)	252 (56.00)	14 (33.33)	238 (58.33)	9.870	0.007
Moderate (35–38)	70 (15.55)	9 (21.42)	61 (14.95)		
Severe (<35)	128 (28.44)	19 (45.23)	109 (26.71)		
CONUT score	4.0 (2.0, 7.0)	6.0 (4.0, 8.0)	4.0 (1.0, 7.0)	−3.402	<0.001
CONUT classification					
Normal (0–1)	112 (24.89)	3 (7.14)	109 (26.71)	14.482	<0.001
Moderate (2–4)	146 (32.44)	10 (23.81)	136 (33.33)		
Severe (5–12)	192 (42.67)	29 (69.04)	163 (39.95)		
HALP score	23.5 (14.8, 41.8)	16.4 (11.8, 25.4)	24.4 (15.3, 43.0)	−3.389	<0.001

**Table 4:** A comparison of echocardiographic findings in patients with IE.

Indicators	Overall (*n* = 450)	Non-survivors (*n* = 42)	Survivors (*n* = 408)	*Z*/χ^2^	*p*-value
LVEF, %	62.7 (59.9, 65.6)	62.0 (59.0, 63.7)	62.7 (60.0, 65.6)	−1.428	0.153
LVEDD, mm	55.0 (50.0, 61.0)	56.5 (52.0, 62.0)	55.0 (49.2, 61.0)	−1.633	0.102
Maximal vegetation diameter, ≥10 mm	153 (34.00)	13 (30.95)	140 (34.31)	0.192	0.661
Valvular stenosis (moderate or severe)	28 (6.22)	2 (4.76)	26 (6.37)	0.169	0.681
Valvular regurgitation (moderate or severe)	32 (7.11)	3 (7.14)	29 (7.11)	0.000	0.993
Valvular prolapse, Yes	131 (29.11)	15 (35.71)	116 (28.43)	0.979	0.323
Valvular perforation, Yes	63 (14.00)	9 (21.43)	54 (13.23)	2.123	0.145
Perivalvular abscess, Yes	32 (7.11)	3 (7.14)	29 (7.11)	0.000	0.993

### 3.2 Assessment of nutritional status

The prevalence of malnutrition risk among patients with IE varied according to the nutritional assessment index used (Table 3). Based on the PNI threshold, 43.99% of patients were at risk of malnutrition, with 28.44% classified as having moderate-to-severe nutritional risk. In contrast, the CONUT score identified 75.11% of patients as being at risk of malnutrition, of whom 42.67% were classified as having severe nutritional risk. In addition, 28% of patients were classified as having severe nutritional risk by both assessment tools.

**Figure 2:**
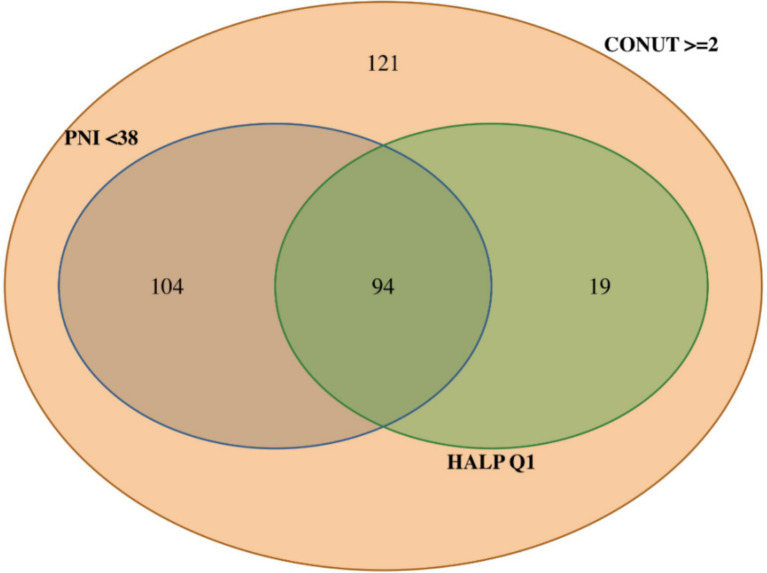
Venn diagram of patients with nutritional risk.

Venn analysis showed that 94 patients met all three nutritional risk definitions, 104 met both PNI and CONUT definitions without low HALP, 19 met the CONUT and low-HALP definitions without PNI-defined malnutrition risk, 121 met CONUT criteria alone, and 112 met none of these definitions. Because HALP has no universally accepted threshold for defining malnutrition risk, the lowest quartile was used only as a descriptive low-HALP category (Figure 2).

**Figure 3:**
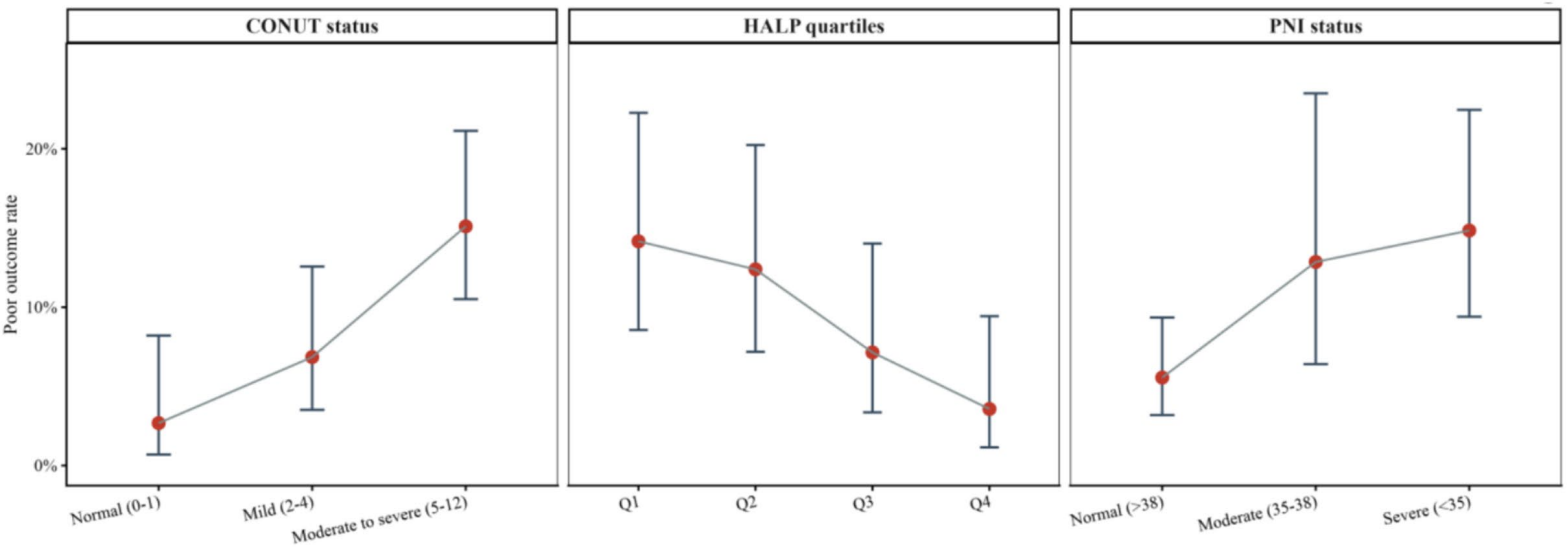
Observed mortality rates across nutritional groups.

**Figure 4:**
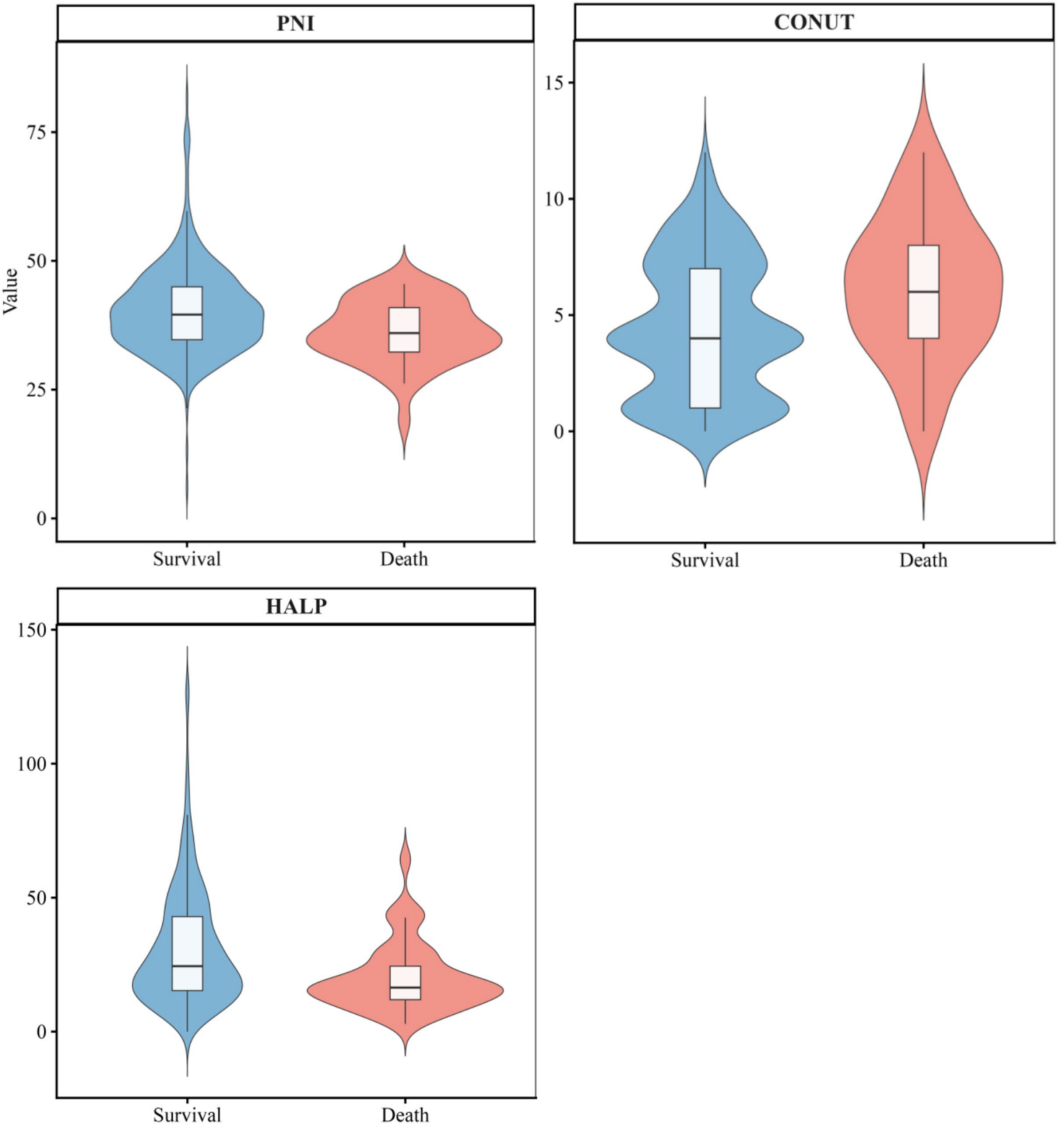
Distribution of nutritional markers by outcome group.

In-hospital all-cause mortality rates differed across nutritional status groups. Patients classified as having moderate-to-severe nutritional risk by both the PNI and CONUT scores had significantly higher in-hospital all-cause mortality rates than patients with normal nutritional status (Figure 3). The proportions of patients identified as being at risk of malnutrition by different nutritional indicators also differed significantly between the non-survivor and survivor groups (Figure 4, Table 3). The median CONUT score was significantly higher in non-survivors than in survivors (*Z* = −3.402, *p* < 0.001), whereas the median PNI and HALP scores were significantly lower in non-survivors (Table 3).

**Figure 5:**
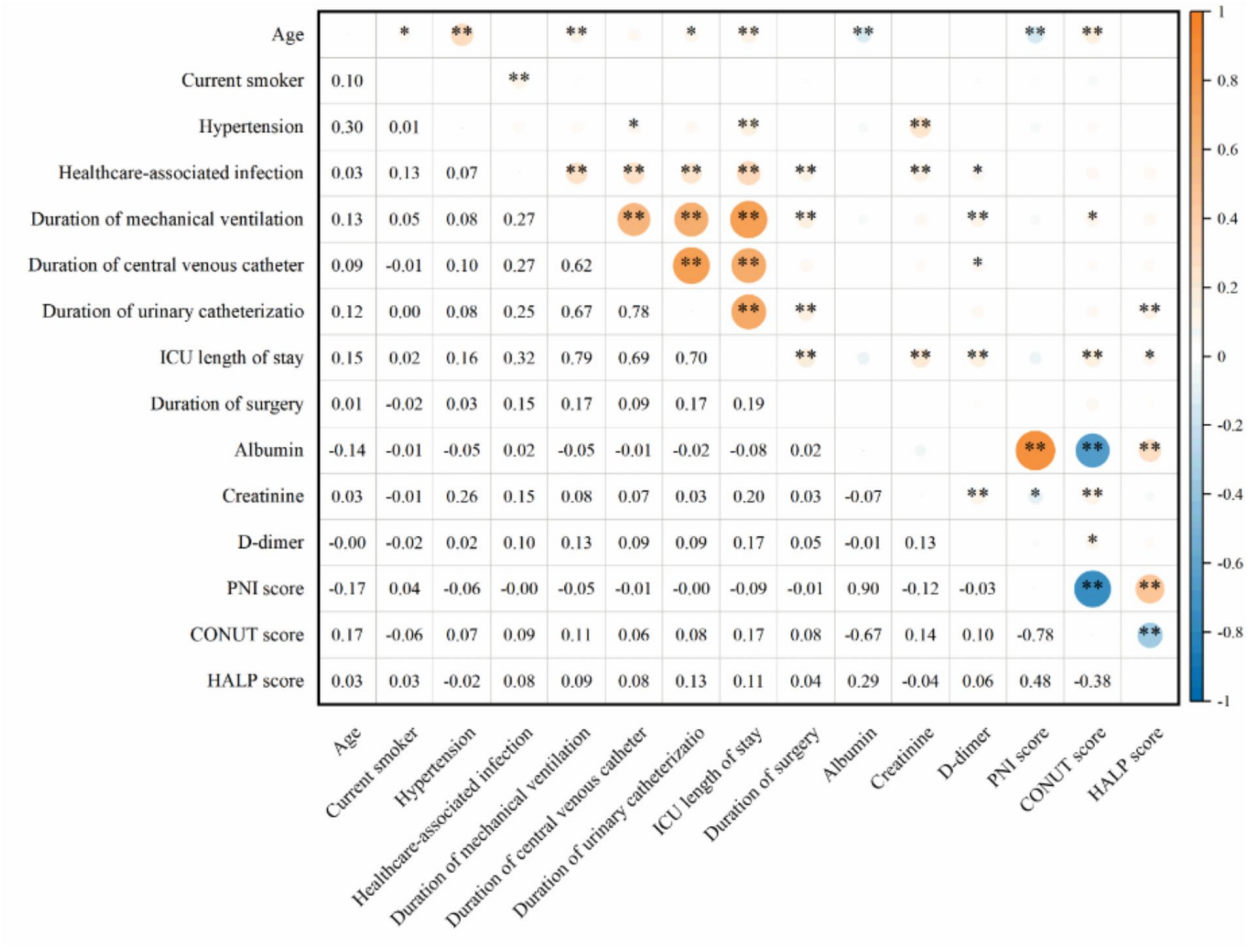
Correlation matrix between clinical parameters and nutritional indicators in patients with IE.

Spearman’s correlation analysis (Figure 5) showed moderate correlations among the three nutritional assessment indices. The CONUT score was negatively correlated with both the PNI and HALP scores (*r* = −0.78 and −0.38, respectively; *p* < 0.001). This pattern supports the internal consistency of these nutritional assessment tools. The strong correlations involving albumin were expected, as albumin is included in the calculation of all three indices.

### 3.3 The predictive value of nutritional scores for in-hospital all-cause mortality

**Figure 6:**
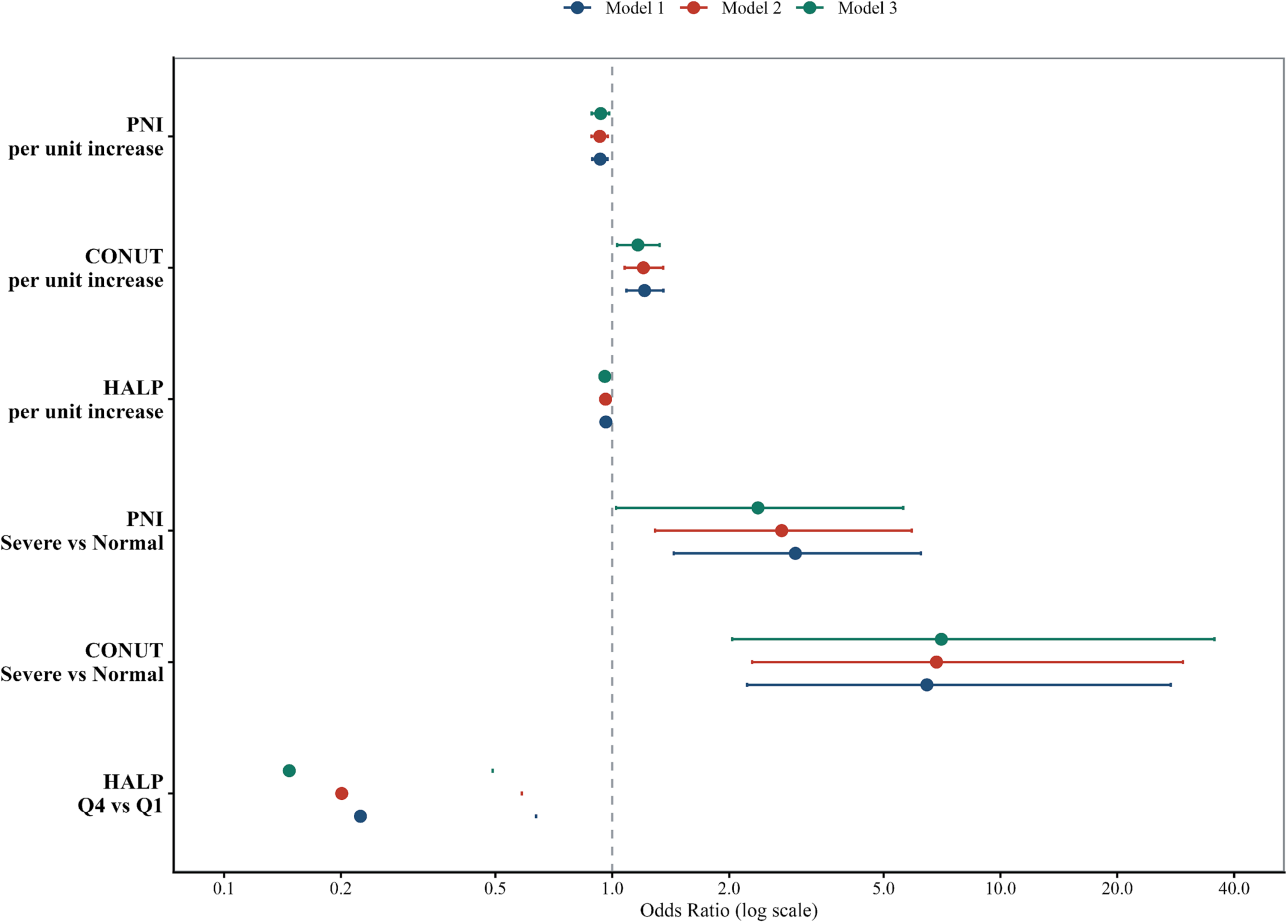
Multi-model nutritional index forest plot.

**Figure 7:**
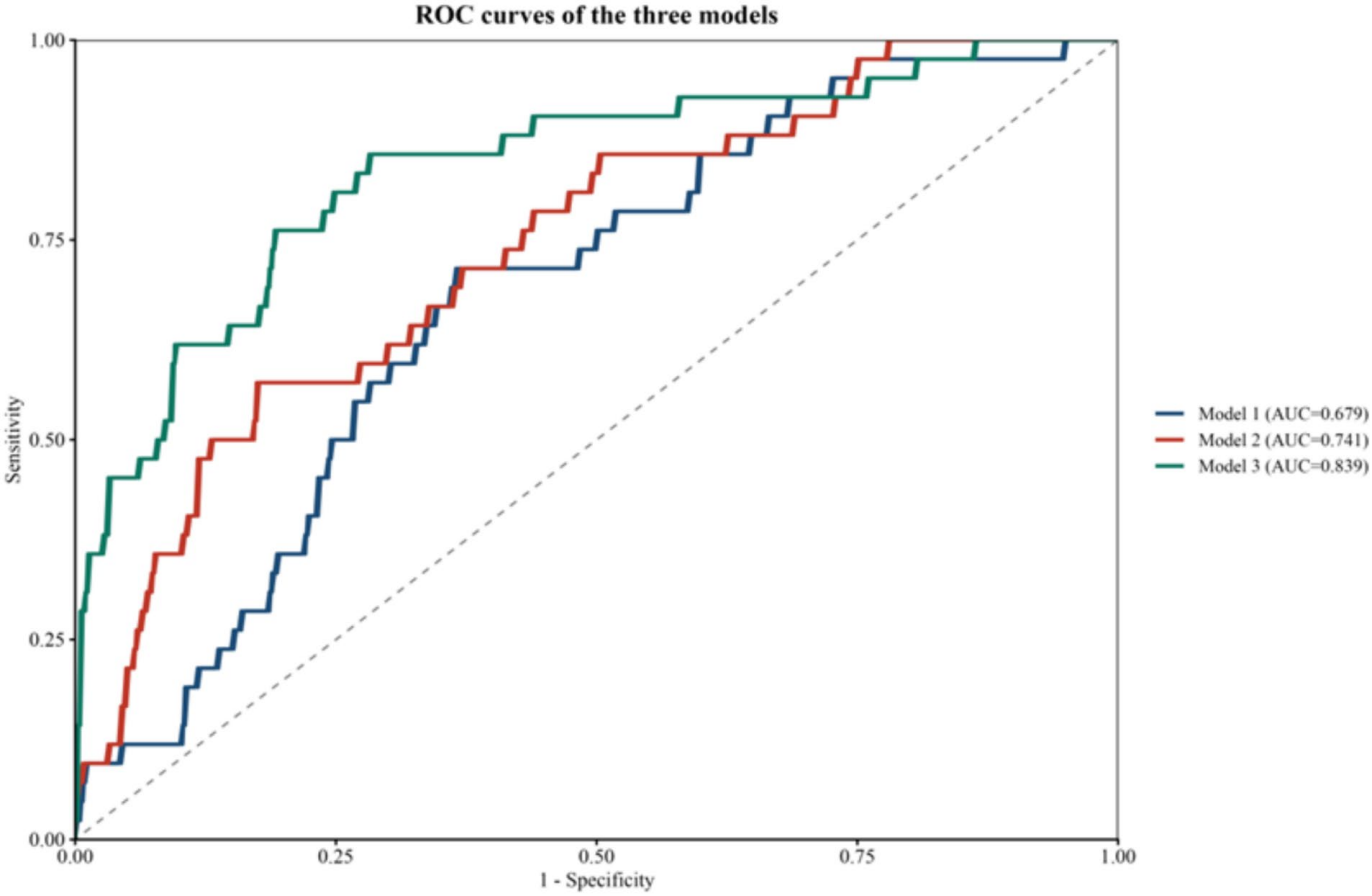
ROC curve.

To further examine the association between nutritional assessment indices and postoperative in-hospital all-cause mortality in patients with IE, three stepwise logistic regression models were constructed (Table 5), and a multi-model forest plot was generated (Figure 6). When the three indices were analyzed as continuous variables, all remained significantly associated with in-hospital all-cause mortality in the models (*p* < 0.05). In the fully adjusted Model 3, which accounted for demographic factors and clinical surgical characteristics, each one-unit increase in PNI was associated with a 7% lower risk of in-hospital all-cause mortality (OR = 0.93, 95% CI: 0.89–0.98, *p* = 0.009). Similarly, each one-unit increase in the HALP score was associated with a 4% lower risk of in-hospital all-cause mortality (OR = 0.96, 95% CI: 0.93–0.98, *p* = 0.001), whereas each one-unit increase in the CONUT score was associated with a 16% higher risk of in-hospital all-cause mortality (OR = 1.16, 95% CI: 1.03–1.32, *p* = 0.017). The performance of the nutritional assessment prediction model was further evaluated using ROC analysis (Figure 7). The ROC curve yielded an AUC of 0.839 (95% CI, 0.768–0.909). Model 3 also had a Brier score of 0.063, which was below 0.25 and lower than those of Model 1 (Brier score = 0.081) and Model 2 (Brier score = 0.077), indicating good discrimination and acceptable predictive accuracy.

**Table 5:** Comparison of multivariate logistic regression models assessing the impact of nutritional status on in-hospital all-cause mortality in patients with IE.

Nutritional parameter	Model 1		Model 2		Model 3	
	OR (95% CI)	*p*-value	OR (95% CI)	*p*-value	OR (95% CI)	*p*-value
PNI score	0.93 (0.89–0.97)	0.002	0.93 (0.88–0.97)	0.002	0.93 (0.89–0.98)	0.009
CONUT score	1.21 (1.09–1.35)	<0.001	1.20 (1.08–1.35)	0.001	1.16 (1.03–1.32)	0.017
HALP score	0.96 (0.94–0.98)	0.002	0.96 (0.94–0.98)	0.001	0.96 (0.93–0.98)	0.001
PNI						
Normal	reference		reference		reference	
Moderate	2.51 (1.00–6.00)	0.041	2.40 (0.94–5.91)	0.059	2.53 (0.85–7.17)	0.085
Severe	2.96 (1.44–6.24)	0.003	2.73 (1.29–5.91)	0.009	2.37 (1.02–5.62)	0.045
*P* for trend	0.003		0.008		0.044	
CONUT						
Normal	reference		reference		reference	
Moderate	2.67 (0.79–12.13)	0.143	3.07 (0.89–14.14)	0.100	3.07 (0.77–16.63)	0.142
Severe	6.46 (2.23–27.46)	0.003	6.84 (2.29–29.54)	0.002	7.04 (2.04–35.59)	0.006
*P* for trend	<0.001		<0.001		0.002	
HALP						
Q1	reference		reference		reference	
Q2	0.86 (0.39–1.85)	0.695	0.85 (0.38–1.90)	0.699	0.72 (0.29–1.77)	0.476
Q3	0.47 (0.18–1.11)	0.094	0.52 (0.20–1.29)	0.167	0.45 (0.15–1.26)	0.138
Q4	0.22 (0.06–0.64)	0.010	0.20 (0.06–0.58)	0.006	0.15 (0.03–0.49)	0.004
*P* for trend	0.003		0.003		0.002	

Model 1: unadjusted. Model 2: adjusted for age, sex, body mass index (BMI), smoking status, alcohol intake, hypertension, diabetes mellitus, and chronic kidney disease. Model 3: further adjusted for healthcare-associated infection, duration of hemodialysis, duration of mechanical ventilation, duration of central venous catheterization, duration of urinary catheterization, ICU length of stay, surgical duration, and type of surgery.

To better assess the independent prognostic value of nutritional indicators, we constructed an additional model that included only preoperative variables. In the new preoperative-only sensitivity model, PNI remained inversely associated with in-hospital mortality (OR = 1.135, *p* = 0.042), HALP remained protective (OR = 0.96, *p* = 0.024); however, CONUT lost its independent prognostic value (Table 6). The ROC curve yielded an AUC of 0.742 (95% CI, 0.658–0.827).

**Table 6:** Logistic regression model for in-hospital all-cause mortality in patients with IE (preoperative variables).

Preoperative variables	B	SE	Wald χ^2^	*p*-value	OR (95% CI)
Constant	−5.452	2.252	5.86	0.015	
Age, years	0.016	0.014	1.429	0.232	1.016 (0.990–1.044)
Current smoker	0.826	0.380	4.714	0.030	2.284 (1.084–4.815)
Hypertension	0.591	0.396	2.220	0.136	1.805 (0.830–3.926)
Hemoglobin, ×10^9/L	−0.001	0.011	0.004	0.948	0.999 (0.978–1.021)
Albumin, g/L	−0.095	0.061	2.438	0.118	0.909 (0.806–1.025)
Creatinine, μmol/L	0.001	0.001	1.103	0.294	1.001 (0.999–1.003)
D-dimer, mg/L	0.044	0.024	3.380	0.066	1.045 (0.997–1.095)
PNI score	0.126	0.065	3.723	0.042	1.135 (0.998–1.290)
CONUT score	0.164	0.105	2.439	0.118	1.178 (0.959–1.446)
HALP score	−0.038	0.017	5.077	0.024	0.962 (0.931–0.995)

### 3.4 Incremental predictive value and internal validation of the nutritional scores

To quantify the incremental prognostic value of the nutritional indices beyond established preoperative clinical variables, we constructed a preoperative baseline model and sequentially added the PNI, CONUT, and HALP scores. Using the preoperative baseline model as the reference (AUC 0.674, 95% CI 0.586–0.761), the addition of each nutritional score improved discrimination: the AUC increased to 0.722 with PNI, 0.712 with CONUT, and 0.735 with HALP, while the exploratory combined model reached 0.741. The likelihood-ratio tests for the addition of PNI, CONUT, HALP, and all three scores were each statistically significant (all *p* ≤ 0.002), and the combined model also showed a significant improvement in AUC versus baseline by the DeLong test (*p* = 0.039) (Table 7).

**Table 7:** Incremental predictive value and bootstrap internal validation of nutritional scores added to the preoperative baseline model.

Model	Apparent performance					Optimism-corrected	
	AUC	95% CI	ΔAUC	DeLong *p*	LR *p*	AUC	Brier
Baseline preoperative model	0.674	0.586–0.761	NA	NA	NA	0.603	0.086
Baseline + PNI	0.722	0.651–0.794	0.049	0.059	0.002	0.657	0.085
Baseline + CONUT	0.712	0.634–0.790	0.038	0.212	0.001	0.647	0.084
Baseline + HALP	0.735	0.654–0.816	0.062	0.053	<0.001	0.676	0.084
Baseline + PNI + CONUT + HALP	0.741	0.664–0.819	0.068	0.039	0.001	0.672	0.085

Given the relatively limited number of in-hospital deaths (*n* = 42), we performed bootstrap internal validation with 1,000 resamples to assess the potential for model overfitting. The optimism-corrected AUCs were 0.603 for the baseline model, 0.657 for baseline + PNI, 0.647 for baseline + CONUT, 0.676 for baseline + HALP, and 0.672 for the combined model, with correspondingly low Brier scores (0.084–0.086) across all models (Table 7).

ΔAUC, change in AUC relative to the baseline model; LR P, *p* value of the likelihood-ratio test; NA, not applicable. Optimism-corrected estimates were obtained by bootstrap internal validation with 1,000 resamples. The combined model also showed a significant AUC improvement versus the baseline model by the DeLong test (*p* = 0.039).

## 4 Discussion

Previous epidemiological studies have reported all-cause mortality rates in IE ranging from 6 to 30%. Consistent with the clinical severity of IE, a multicenter registry of the European Society of Cardiology (ESC) from 2016 to 2018 reported an in-hospital mortality rate of 17.1%, which increased to 32% among surgical patients (32). Similarly, ICE-PCS, one of the largest international IE registries, reported an overall mortality rate of 18% (33). In the present cohort of 450 surgically treated patients with IE, the in-hospital all-cause mortality rate was 9.33%, which is lower than many previously reported estimates. This may be partly explained by recent institutional improvements, including advances in surgical techniques, pathogen-guided antimicrobial therapy, multidisciplinary endocarditis team management, and improved critical care. Despite this relatively favorable survival rate, many patients had compromised preoperative nutritional status. Malnutrition risk was present in 44.00% of patients according to the PNI and in 75.11% according to the CONUT score. This high sensitivity of the CONUT score aligns with recent findings in acute cardiovascular cohorts (34). The difference in prevalence reflects the distinct diagnostic focus of each tool. Still, the overlap between them is clinically important: 28% of patients were classified as having moderate-to-severe nutritional risk by both PNI and CONUT. This overlapping high-risk subgroup may represent the most appropriate target for early and focused nutritional intervention.

In serious inflammatory and infectious conditions, malnutrition has been consistently associated with adverse outcomes, including in cancer (35), large-vessel acute ischemic stroke (13), and severe sepsis (36). Systemic nutritional deficits in these settings often indicate higher mortality risk, longer hospitalization, and greater healthcare burden. The present study supports the prognostic value of the PNI, CONUT, and HALP indices for in-hospital all-cause mortality in surgically treated patients with IE. After full multivariable adjustment, higher PNI and HALP scores remained protective prognostic indicators. The correlations among these indices, especially the strong negative correlation between CONUT and PNI, support their shared ability to reflect host immunonutritional status. At the same time, each index captures a different dimension of systemic pathology in IE. The shared albumin and lymphocyte components of PNI and CONUT reflect protein catabolism and immune suppression driven by severe systemic inflammation. In addition, CONUT incorporates total cholesterol, which may reflect lipid homeostasis and bioenergetic reserve (37, 38). In this cohort, non-survivors had significantly higher CONUT scores than survivors, highlighting the potential relevance of energy reserve and dyslipidemia in the hypermetabolic inflammatory state of IE. The HALP score provides a broader physiological perspective by integrating hemoglobin, albumin, lymphocytes, and platelets. As recently demonstrated by Hancıoğlu et al. (39), the HALP score strongly correlates with in-hospital mortality in IE because it captures not only nutritional deficiency but also the hematological exhaustion and microcirculatory derangements characteristic of severe endocarditis.

When considering how each scoring system may be used clinically in this IE cohort, their optimal applications may differ. The CONUT score identified the largest proportion of patients at nutritional risk, making it useful as an early screening tool for capturing vulnerable individuals. The HALP score, meanwhile, showed a strong independent association with in-hospital all-cause mortality in the fully adjusted model, suggesting potential value for more refined mortality risk stratification. The PNI also remained an independent predictor and is easy to calculate. Beyond confirming the independent association of each nutritional index with in-hospital mortality, we further examined whether these scores provide incremental value over routinely available preoperative variables. Because the PNI and CONUT scores were strongly correlated (*r* = −0.78), entering them into a single model would have introduced substantial collinearity; we therefore added each score to the preoperative baseline model separately, an approach consistent with recommendations for handling correlated predictors in clinical prediction modeling (40). Each score significantly improved discrimination over the baseline model, with HALP yielding the largest gain. However, an exploratory model combining all three indices provided little additional benefit over the best single score—a pattern expected given their shared albumin component and mutual correlation. This suggests that a single, readily obtainable nutritional index may be sufficient for preoperative risk stratification, avoiding the redundancy that accompanies the co-entry of collinear scores.

Notably, while objective nutritional indices demonstrated strong prognostic capacity, traditional IE-specific clinical variables—including echocardiographic vegetation size (≥10 mm) and specific microbiological profiles (e.g., Staphylococcus spp. vs. Streptococcus spp.)—did not emerge as independent predictors of in-hospital all-cause mortality in our surgical cohort. Previous studies have demonstrated that *Staphylococcus aureus* and large vegetations promote embolic complications and correlate with long-term adverse events (41, 42). However, their impact on short-term, postoperative survival is often subordinate to the patient’s baseline physiological reserve. Acute operative stress imposes a significant load on the host’s metabolic state. As stated by Eranki et al. (43), predicting acute in-hospital mortality is more successful through the use of perioperative physiological parameters and surgical factors, as opposed to anatomical vegetation characteristics. Our results reinforce this paradigm, suggesting that once the surgical indication is met, the immunonutritional resilience of the host becomes the primary determinant of surviving the acute postoperative phase.

Our findings support the integration of PNI, CONUT, and HALP assessment into routine preoperative evaluation for surgically treated patients with IE. These indices are based on widely available and cost-effective hematological and biochemical parameters that can be obtained within 24 h of admission. For patients at moderate-to-high nutritional risk, early multidisciplinary nutritional support should be considered alongside antimicrobial and surgical treatment. Individualized regimens may include high-calorie, protein-rich preoperative enteral nutrition, careful perioperative micronutrient replacement, and targeted immunonutritional strategies when appropriate, particularly for patients with hypoalbuminemia or lymphopenia.

## 5 Limitation

Despite its clinical relevance, this study has several limitations. First, this was a single-center, 14-year retrospective study, which may have introduced selection bias. Changes in treatment strategies over such a long study period may also have influenced the results. Second, this study included only patients with IE who underwent surgical treatment; patients who were too ill to undergo surgery or who received conservative treatment were not included. Therefore, rates of mortality and nutritional risk in the overall IE population may have been underestimated. Third, the relatively small number of in-hospital deaths (*n* = 42) resulted in a modest events-per-variable ratio and a potential risk of overfitting. Although bootstrap internal validation indicated only limited optimism, these findings require external validation in larger, multicenter cohorts. Future multicenter prospective cohort studies are needed further to validate the clinical utility of these nutritional scores and to determine whether specialized nutritional intervention regimens can improve outcomes in high-risk patients with IE.

## 6 Conclusion

Among patients requiring surgical treatment for IE, systemic nutritional depletion is common. Prognostic models based on routinely available laboratory indices, including the PNI, CONUT, and HALP scores, were independently associated with postoperative in-hospital all-cause mortality. Combining these tools may support rapid baseline risk stratification and help identify vulnerable patients who may benefit from early nutritional intervention. Integrating nutritional assessment into perioperative IE management may help address the interconnected burden of infection, metabolic deterioration, and immune dysfunction, ultimately improving risk-guided care for this high-risk population.

## Data Availability

The original contributions presented in the study are included in the article/supplementary material, further inquiries can be directed to the corresponding author.
